# CRP Enhances the Innate Killing Mechanisms Phagocytosis and ROS Formation in a Conformation and Complement-Dependent Manner

**DOI:** 10.3389/fimmu.2021.721887

**Published:** 2021-08-10

**Authors:** Johannes Zeller, Balázs Bogner, Jurij Kiefer, David Braig, Oscar Winninger, Mark Fricke, Ebru Karasu, Karlheinz Peter, Markus Huber-Lang, Steffen Ulrich Eisenhardt

**Affiliations:** ^1^Department of Plastic and Hand Surgery, Medical Center - University of Freiburg, Faculty of Medicine, University of Freiburg, Breisgau, Germany; ^2^Division of Hand, Plastic and Aesthetic Surgery, University Hospital, Ludwig Maximilian University of Munich (LMU), Munich, Germany; ^3^Institute of Clinical and Experimental Trauma-Immunology, University Hospital of Ulm, Ulm, Germany; ^4^Atherothrombosis and Vascular Biology Laboratory, Baker Heart and Diabetes Institute, Melbourne, VIC, Australia; ^5^Department of Cardiometabolic Health, University of Melbourne, Melbourne, VIC, Australia

**Keywords:** innate immunity, host defense, C-reactive protein, reactive oxygen species, complement system, complement inhibition

## Abstract

Phagocytosis and the formation of reactive oxygen species (ROS) in phagocytic leukocytes are an effective killing mechanism of the innate host defense. These cellular processes of innate immunity function in a complex interplay with humoral factors. C-reactive protein (CRP) in its activated, monomeric isoform (mCRP) has been shown to activate immune cells *via* the classical complement pathway. We investigated the complement-dependent effects of monomeric CRP (mCRP) on neutrophils and monocyte subtypes using complement-specific inhibitors by both flow cytometry and confocal fluorescence microscopy. We demonstrate that CRP-induced ROS generation is a conformation-specific and complement-dependent process in leukocyte subsets with classical monocytes as the primary source of ROS amongst human monocyte subsets. Elucidation of this complex interplay of CRP and complement in inflammation pathophysiology might help to improve anti-inflammatory therapeutic strategies.

## Introduction

The innate immune system represents the foundation of host defense with phagocytotic leukocytes as its mainstay. The most abundant phagocytic cell subtype in human whole blood is the polymorphonuclear neutrophil (PMN). Monocytes represent the second most frequent cell line with pro-inflammatory classical monocytes (CD14++CD16-) representing the vast majority and patrolling non-classical monocytes (CD14+CD16++) as a second subpopulation comprising up to 10% of all circulating monocytes (1). Despite the high plasticity and heterogeneity of monocytes, the subset identification is generally based on the relative expression of CD14 and CD16 (2, 3). Therefore, most studies investigating ROS focus on neutrophils. However, this oxygen-dependent killing mechanism of invading pathogens is not limited to one leukocyte subtype; monocytes and macrophages share the same host defense mechanism. In addition to the cellular innate immune response, the humoral components represent the second principal mode of action. C-reactive protein (CRP) has been recognized as the archetype acute phase reactant in both sterile and non-sterile immune response (4). It represents a critical factor in amplifying an unspecific response to tissue damage: Acting as a primary initiator of the classical complement pathway, CRP aggravates the inflammatory response mainly in a complement- dependent manner. Complement initiation culminates in C3 hydrolysis and active C3b cleaves C5 into C5a and C5b fragments. *Via* the complement C5a receptor C5aR1 (CD88), this anaphylatoxin exerts a strong inflammatory effect on neutrophils and monocytes (5). Besides protective functions, massively increased ROS production during sepsis may also have contrary effects and lead to increased host tissue damage. Here, we investigated the effects of CRP and complement-dependent cell activation on ROS generation and phagocytosis in human leukocyte subsets.

## Material and Methods

### Dialysis and Preparation of CRP

Human pCRP was purchased from life diagnostics (West Chester, PA, USA) and preparation was performed as described before (6). In brief, human pentameric CRP was dialyzed against Dulbecco’s phosphate buffered saline (DPBS) supplemented with 0.9 mM calcium chloride (CaCl_2_) and 0.49 mM magnesium chloride (MgCl_2_). Dialyzed pCRP was tested as described before (6, 7). Monomeric CRP was generated by treating pCRP with 8 M urea and 100 µM EDTA for one hour at 37°C and following dialysis against 25 mM Tris-HCl (pH 8.3) overnight at 4°C (8). The protein concentration was determined after each dialysis and dissociation procedure by a benchtop fluorometer (Qubit^®^ 3.0 Fluorometer, Invitrogen™ by life technologies™, Carlsbad, CA, USA).

### Complement System Inhibitors

The complement system inhibitors Compstatin, PMX53, and OmCI were used to evaluate the impact of the complement system in the activation of leukocyte subsets. The cyclic hexapeptide PMX53 (sequence Ace-Phe-[Orn-Pro-dCha-Trp-Arg]) was used to antagonize C5aR1 activation as described before in a concentration of 10 µg/mL and was added 15 min prior to exposure to the relevant agents (5, 9, 10). Compstatin inhibits the activation of both the classical and alternative complement pathways by selectively binding to native C3 (11). It was used as described before in 50 µM concentration in whole blood (12). OmCI is an effective C5 inhibitor in pigs and humans isolated from the soft tick African hut tampan (*Ornithodoros moubata*) *(* 13).

### Red Blood Cell Lysis Solution

Red blood cell (RBC) lysis solution was prepared as described before (3). In short, 80.2 g ammonium chloride (NH_4_Cl), 8.4 g sodium bicarbonate (NaHCO_3_), and 126 mL of a 100 mM EDTA solution were added to 874 mL milli-Q™ water (1000 mL total end volume). This stock solution (tenfold concentration of the used RBC lysis solution) was kept at 4°C. Onefold solutions were prepared fresh every seven days and adjusted to pH 7.8 and kept at 4°C as well.

### Antibodies

The antibodies used for differentiation in the described method were anti-human CD14 Pacific Blue^®^ (Clone: M5E2), anti-human CD16 phycoerythrin-cyanine 7 (PE-Cy 7, Clone: 3G8), and anti-human HLA-DR allophycocyanin and fluorescein isothiocyanate (APC, FITC, Clone: TU36), and negative lineage markers anti-human CD2 PE (Clone: RPA-2.10), anti-human CD15 PE (Clone: VIMC6), anti-human CD19 PE (Clone: HIB19), anti-human CD56 PE (Clone: MY31), and anti-human NKp46 PE (Clone: BAB281) from BD Biosciences, Franklin Lakes, NJ, USA. For fluorescence compensation, the VersaComp antibody capture bead kit from Beckman Coulter, Inc., Brea, CA, USA was used. All flow cytometry-based assays were analyzed on BD LSR Fortessa™ cell analyzer.

### Blood Sampling

Human whole blood was drawn freshly from healthy and informed donors without drug intake for at least two weeks for all experiments described. Hereby, the guidelines of the local ethics committee were ensured. Whole blood was drawn from the median cubital vein into heparinized vacutainers (16 IU/mL, Sarstedt AG & Co. KG, Nümbrecht, Germany). The first 10 mL were discarded to avoid unspecific cell activation. The time between the blood sampling and processing was as short and reproducible as possible (< 30 min) to exclude unspecific activation due to prolonged latency (14). For each sample, 100 μL of heparinized whole blood was transferred into sterile 2 mL polypropylene collection tubes (Biozym Scientific GmbH, Hessisch Oldendorf, Germany) for further testing.

### Gating Strategy for Reactive Oxygen Species in Whole Blood

Monocyte subtypes were defined by CD14 and CD16 expression as described before (3). Briefly, classical monocytes were suggested expressing CD14++ and CD16-, whereas non-classical monocytes express CD16++ and CD14+. Intermediate monocytes (CD14+ and CD16+) were disregarded for this study. Further, HLA-DR expression was used to differentiate CD16-positive neutrophils and natural killer cells from non-classical monocytes. Cells expressing no HLA-DR (neutrophils and NK-cells) were excluded.

### Flow Cytometry for Reactive Oxygen Species in Whole Blood

100 µL heparinized human whole blood was incubated with 50 µg/mL mCRP or pCRP, 10 µg/mL LPS and 20 µM ADP for 180 min periods at 37°C, 5% CO_2_. After 150 min incubation, DHE was added to a final concentration of 10 µg/mL. ROS detection in this setting is prone to temperature fluctuations and aldehyde fixation. All samples were lysed with 2 mL RBC lysis buffer at pH 7.4 and room temperature for 5 min in the dark. Cell suspensions were then pelleted (400 x g, 5 min at RT) and resuspended in 100 µL FACS buffer. Anti-human CD16 PE-Cy7, CD14 Pacific blue^®^, and HLA-DR APC were added 1:100 v/v and incubated for 15 min at RT and dark. Finally, 400 µL of FACS buffer was added and the samples subsequently analyzed by flow cytometry. DHE fluorescence in lymphocytes was set as a negative control. CellROX™ Deep Red (Thermo Fisher, Waltham, MA, USA) was used as a second ROS detecting agent in whole blood as described by the producer. A final concentration of 2.5 µM was used for 30 min and fixed with 4% paraformaldehyde. Whole blood was treated as described above. Antibodies were customized to fit the CellROX Deep Red fluorescence: Anti-human CD16 PE-Cy7, CD14 Pacific blue^®^, and HLA-DR FITC and negative lineage markers anti-human CD2 PE (Clone: RPA-2.10), anti-human CD15 PE (Clone: VIMC6), anti-human CD19 PE (Clone: HIB19), anti-human CD56 PE (Clone: MY31), and anti-human NKp46 PE (Clone: BAB281) were added 1:100 v/v and incubated for 15 min at RT and dark.

### Phagocytosis Target Staining With Fluorescein Isothiocyanate

Phagocytosis targets were tagged with fluorescein isothiocyanate (FITC) for assessment of phagocytosis in whole blood samples by flow cytometry and confocal fluorescence microscopy. Particles were conjugated in 0.1 M sodium carbonate buffer, pH 9.0. 84 mg sodium carbonate (Na_2_CO_3_) was dissolved in 10 mL milli-Q^®^ water. The buffer was adjusted to pH 9.0 and filtered through 0.22 µM PVFD filters (Carl Roth GmbH, Karlsruhe, Germany). The FITC stock solution was prepared by dissolving 10 mg FITC (Carl Roth GmbH, Karlsruhe Germany) in 1 mL dimethyl sulfoxide (DMSO). Heat inactivated bacteria and zymosan particles were washed in sodium carbonate buffer and centrifuged at 10,000 x g for 5 min at 4°C and resuspended in 1 mL 0.1 M sodium carbonate buffer. Cell count was measured by OD measurements with the sodium carbonate buffer as the blank and the cell count adjusted to 10 (15) cells per mL. The FITC concentration was adjusted to 0.05 mg/mL for both bacterial targets and set to 0.5 mg/mL for zymosan. The targets incubated in the dark for 30 min at 600 rpm and RT for FITC tagging. Unbound FITC molecules were removed by four washing steps in PBS supplemented with calcium and magnesium at 10,000 x g for 5 min and 4°C. The fluorescence tagged particles were resuspended in 1 mL PBS, and the cell count was finally measured. Fluorescence tagged targets were used immediately or stored at -20°C for maximal 30 days.

### Confocal Fluorescence Microscopy of Phagocytosis in Whole Blood

Phagocytosis in whole blood samples was assessed by flow cytometry and verified by confocal immunofluorescence microscopy. Human whole blood samples were incubated as described above. For confocal immunofluorescence microscopy, cells were counted after RBC lysis and adjusted. To detect phagocytosis, cells were stained and washed again and immobilized on ibidi^®^ µ-slides VI ^0.4^ channel slides. After one hour, the vessels were thoroughly washed with DPBS supplemented with calcium to remove debris and adherent cells were fixed with 4% paraformaldehyde in DPBS for 5 min at RT. After fixation, cells were washed twice with DPBS and permeabilized with cold DPBS, Triton X-100 0.3%, 1% BSA, at RT for 5 min. Cells were washed again and incubated with 300 nM 4,6-diamidino-2-phenylindole (DAPI) (Sigma-Aldrich) for five minutes and washed. F-actin was stained with phalloidin-Alexa Fluor 647 (Thermo Fisher, Waltham, MA, USA) following the manufacturer’s instructions, washed, and mounting medium (ibidi GmbH, Planegg, Germany) was finally applied to each channel. Image acquisition was performed by confocal immunofluorescence microscopy (Zeiss LSM 980, Carl Zeiss Microscopy GmbH, Jena, Germany) and processed with microscopy image analysis software Imaris (Bitplane Oxford Instruments, Belfast, UK). The cells were analyzed using three laser lines: Multiline Argon (458, 488, 514 nm), helium-neon laser (561 nm and 633 nm), and a UV diode (405 nm). For each picture, laser intensity and amplifier gain were adjusted to avoid pixel saturation. To avoid spectral overlap, sequential detection was performed, and each fluorophore used was exited independently. The resolution of each picture was at least 1024 x 1024 pixels. Magnification of the objective was x20 and x63, with a pinhole diameter of 1 airy unit. DAPI was excited at 405 nm, fluorescein complex at 488 nm, and phalloidin-Alexa Fluor™ 647 at 650 nm.

### Statistical Analysis

All ROS data are presented as mean and standard error of the mean (SEM) of the mean fluorescence intensity of the relevant fluorescence if not stated otherwise. 2-hydroxyethidium fluorescence was used as a surrogate for ROS generation in each subset and compared to the appropriate baseline measurements of the unstimulated controls using analysis of variance (ANOVA) and Tukey *post hoc* test. Phagocytosis data is presented as FITC positive cells per all cells of the relevant subtype in percent and is depicted as mean and SEM. Each time-point is compared to the appropriate control measurements stated in the figures and text using ANOVA and Tukey *post hoc* test. P values < 0.05 were considered statistically significant. All statistical analyses were performed using GraphPad Prism 9 for Mac (GraphPad Software, LLC Ltd., La Jolla, CA).

## Results

### Subset-Specific ROS Generation in Leukocytes Is Detectable in Human Whole Blood Samples

Human whole blood samples were prepared as described and analyzed by flow cytometry. After lysis and washing, cells resuspended in FACS buffer were differentiated by size and internal complexity and CD14, CD16, and HLA-DR expression as described before (3). The gating strategy shown enables the distinct detection of ROS generation in the two primary ROS generating leukocyte subsets, classical monocytes and neutrophils, and non-classical monocytes (**Supplementary Figure 1**). An unstimulated control (blue) was clearly differentiable from a positive control stimulated with LPS (red) in all three subsets.

### mCRP But Not pCRP Induces ROS Generation in Human Leukocytes

**Figure 1:**
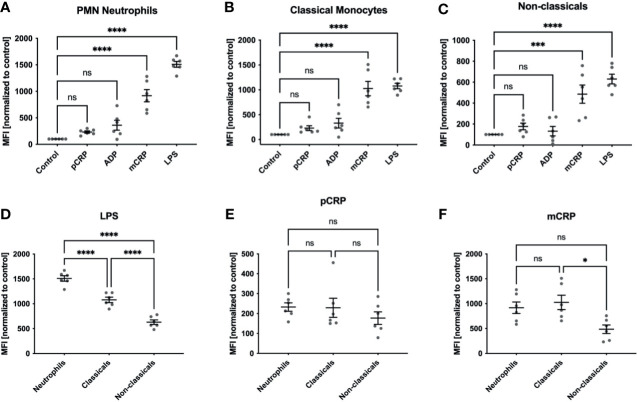
ROS formation in human whole blood samples. ROS formation in neutrophils **(A)**, classicals **(B)** and non-classicals **(C)** was assessed after incubation with 50 µg/mL pCRP and mCRP, 20 µM ADP, 10 µg/mL LPS, respectively, or stayed unstimulated (control). We observed a significant increase in ROS generation in neutrophils and both monocyte subsets when treated with 25 µg/mL mCRP, but not 50 µg/mL pCRP ***P < 0.001 and **** < 0.0001, **(A–C)**. Cells incubated with 20 µM ADP failed to raise ROS production significantly over the control level. LPS served as a positive control and showed the most substantial increase in ROS generation in all three leukocyte subsets (****P < 0.0001). When all three subsets were compared for agent-specific ROS generation, we found classical monocytes to produce a higher respiratory burst than in the non-classical subset. This difference was found in both LPS and mCRP-stimulated cells **(D, F)**. However, LPS induced the strongest respiratory burst in the neutrophil subset (ns not significant, *P < 0.05, **** and P < 0.0001, **(D)**. For pCRP-stimulated cells no significant difference was found **(E)**. Results are presented as means and S.E.M., P values were calculated with ANOVA and *Tukey post hoc* test, n = 6, ns, not significant; *** and ****P < 0.001 and < 0.0001.

We observed a significant increase in ROS generation in neutrophils and both monocyte subsets when treated with 25 µg/mL mCRP and measured by dihydroethidium (DHE) in flow cytometry (*** P < 0.001 and **** < 0.0001, **Figures 1A–C**). Cells incubated with 50 µg/mL pCRP showed no increase in ROS production in any leukocyte subtype. 20 µM ADP failed to raise ROS production significantly over the control level. LPS served as a positive control and showed the most substantial increase in ROS generation in all three leukocyte subsets (**** P < 0.0001). Further, DHE fluorescence as a surrogate of ROS generation in whole blood was measured in lymphocytes as a negative control (**Supplementary Figure 2A**). Both LPS and mCRP failed to induce ROS generation in lymphocytes, while monocytes and neutrophils showed a significant shift in the relevant fluorescence channel (blue histograms) compared to an unstimulated control (red histograms, **Supplementary Figure 2B**).

### Classical Monocytes Are the Primary Source of ROS Amongst Human Monocyte Subsets

We found classical monocytes to produce a higher respiratory burst than in the non-classical subset with DHE conversion as a surrogate (**Figures 1D–F**). This difference was not dependent on a specific pro-inflammatory agent and was found after both LPS and mCRP incubation (**Figures 1D, F**). However, LPS induced the strongest respiratory burst in the neutrophil subset (ns not significant, * P < 0.05, **** and P < 0.0001).

### CRP-Induced ROS Formation in Classical Monocytes and Neutrophils Depends on Complement Activation

**Figure 2:**
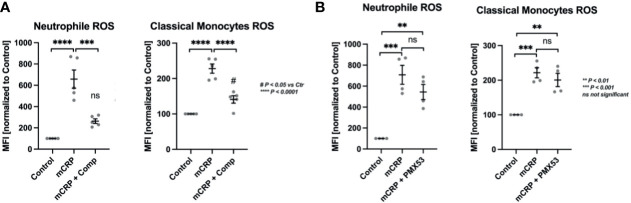
CRP-induced ROS formation depends on complement activation, but not C5aR1 in particular. CRP-induced ROS formation was assessed in the two mainstay cell populations of ROS formation, PMNs and classical monocytes. In whole blood treated with the C3 convertase inhibitor Compstatin, mCRP-stimulated neutrophils failed to produce significant respiratory burst over control levels while classical monocytes did **(A)**. However, Compstatin reduced ROS generation in both subtypes significantly. In neutrophils and monocytes treated with C5aR1 antagonist PMX53, mCRP-induced ROS formation was not reduced significantly compared to the adequate positive control (mCRP) **(B)**. The respiratory burst detected in both populations was still significantly elevated over unstimulated controls in both leukocyte subsets (** P < 0.01 and *** P < 0.001) **(B)**. All results are presented as means and S.E.M. and P values were calculated with ANOVA and *Tukey post hoc* test, n = 5, ns, not significant; ^#^P < 0.05, **, *** and **** P < 0.01, < 0.001, < 0.0001.

The two leukocytic mainstays of ROS formation were further investigated for their complement-dependent mode of action in CRP-induced ROS formation (**Figure 2**). In whole blood treated with Compstatin, a C3 convertase inhibitor, mCRP-stimulated neutrophils failed to produce a significant respiratory burst over control levels while classical monocytes did. However, Compstatin reduced ROS generation in both subtypes significantly (*** P < 0.001 and **** P < 0.0001, respectively) (**Figure 2A**).

### C5aR1 Blockade Does Not Influence CRP-Dependent ROS Formation

In neutrophils and monocytes treated with the C5a receptor 1 antagonist PMX53, mCRP-induced ROS formation was not reduced significantly compared to the relevant control (mCRP). The respiratory burst detected in both populations was still significantly elevated over unstimulated controls (** P < 0.01 and *** P < 0.001) (**Figure 2B**).

### Subtype-Specific Phagocytosis of Common Pathogens Can Be Assessed in a Time-Dependent Manner in Human Whole Blood

The three common target particles Zymosan, heat-killed S. pneumoniae and E. coli were fluorescein isothiocyanate (FITC)-labeled and phagocytosis was assessed by flow cytometry. The three most abundant phagocytic subpopulations are clearly distinguishable as demonstrated representatively with S. pneumoniae-FITC targets (**Supplementary Figure 4A**). Moreover, different time-points are pronounced as represented by Zymosan-FITC phagocytosis in neutrophils at the 5 min (blue histogram) and 10 min (red histogram) time-point (**Supplementary Figure 4B**), which shows time-dependent accumulation all three target particles, e.g., E. coli-FITC in neutrophils at 5, 10, 15, and 20 min (histogram overlay from light to dark-green, **Supplementary Figure 4C**).

### C-Reactive Protein Opsonizes *Streptococcus pneumoniae* and Facilitates the Phagocytosis in Classical Monocytes, But Not in Non-Classical Monocytes and Neutrophils

**Figure 3:**
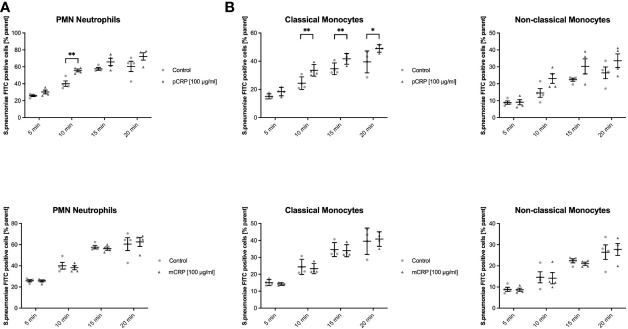
pCRP facilitates phagocytosis of S. pneumoniae-FITC targets by opsonization, while mCRP does not. Phagocytosis by neutrophils **(A)**, classical, and non-classical monocytes **(B)** was assessed by the described flow cytometry protocol. FITC-tagged S. pneumoniae incubated with 100 µg/mL pCRP before exposure to phagocytic cells were engulfed significantly faster and in higher amounts compared to an untreated control in all three subtypes investigated **(A, B)**. mCRP added to the target suspension in the same concentration showed no significant effects on the phagocytosis in all assessed subtypes and time-points **(C, D)**. All results are presented as means and S.E.M. and P values were calculated with ANOVA and *Tukey post hoc* test, n = 4, * and ** P < 0.05 and < 0.01.

Human whole blood assessed for phagocyted S. pneumoniae-FITC targets in leukocyte subsets showed a distinct phagocytotic activity when targets were treated with pCRP. Classical monocytes internalized significantly more S. pneumoniae-FITC targets when treated with pCRP but not with mCRP compared to an untreated control at 10, 15, and 20 min, but not at 5 min. In neutrophils phagocytosis was significantly enhanced by pCRP at all time-point except 5 min. However, all three subtypes showed a tendency towards pCRP-facilitated phagocytosis at every time point analyzed (* *P* < 0.05, and ** < 0.01 and *P* > 0.05, respectively, **Figures 3A–C**).

### mCRP Does Not Opsonize *Streptococcus pneumoniae*

Blood assessed for engulfed S. pneumoniae-FITC targets showed no difference in phagocytotic activity when targets were treated with mCRP compared to untreated controls. All three subsets showed no significant difference in mCRP-treated phagocytosis at any time point analyzed (**Figures 3D–F**).

### CRP Does Not Influence the Phagocytosis of FITC-Tagged *Escherichia coli*, Neither in Monocyte Subsets Nor in Neutrophils

**Figure 4:**
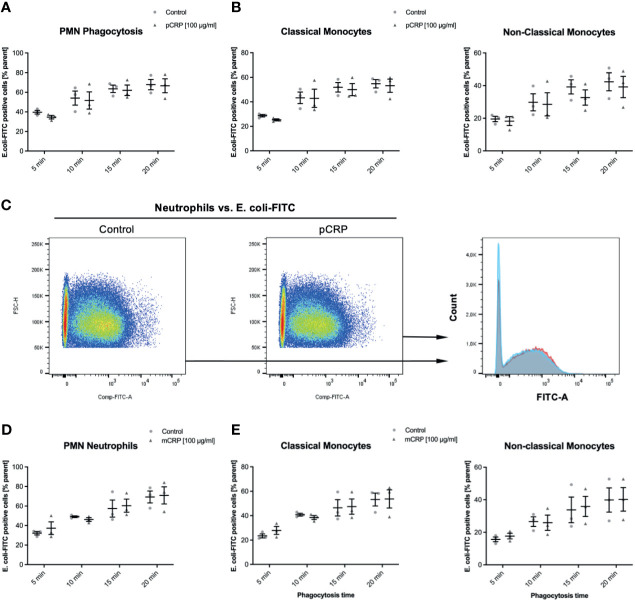
Elimination of E. coli is neither affected by pCRP nor mCRP in whole blood leukocytes. Phagocytosis of E. coli targets was assessed as described. Heat-killed E. coli were treated with either 100 µg/mL pCRP **(A–C)** or mCRP **(D, E)**, respectively. **(C)** shows the final gate in the used flow cytometry gating strategy. Compared to untreated E. coli, both CRP isotypes failed to influence the phagocytosis significantly in any assessed subtype. Results shown in means and S.E.M. and P values were calculated with ANOVA and *Tukey post hoc* test, n = 3, no significant differences.

E. coli targets treated as described above were incubated with 100 µg/mL pCRP and mCRP, respectively. Both CRP isotype treatments did not significantly influence the phagocytosis of E. coli targets in any leukocyte subset (**Figure 4**). 100 µg/mL pentameric CRP compared to an untreated control E. coli-specimen showed no difference in FITC-positive neutrophils at any investigated time-point (**Figures 4A–C**). **Figure 4D** shows representative results at 15 min time-point for the phagocytosis of E. coli in neutrophils. The overlay clarifies the non-significant difference (P > 0.05).

### Zymosan-FITC Is Cleared More Efficiently After pCRP Opsonization in Both Monocyte Subsets, But Not in Neutrophils

**Figure 5:**
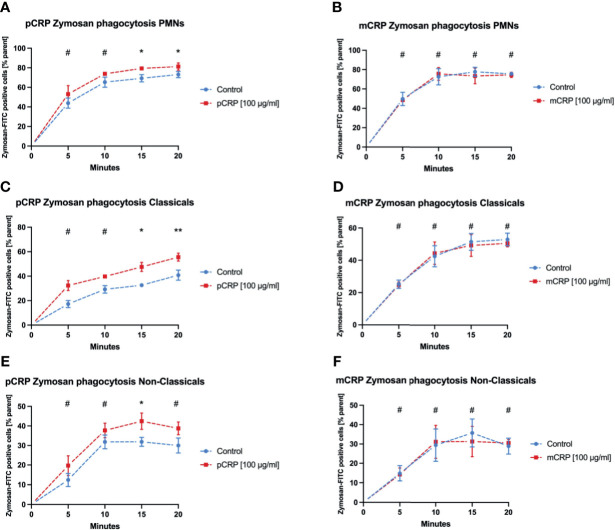
pCRP facilitates phagocytosis of zymosan-FITC targets by opsonization, while mCRP does not. Phagocytosis by neutrophils **(A, B)**, classical monocytes **(C, D)**, and non-classical monocytes **(E, F)** was assessed by the described flow cytometry protocol. pCRP added to FITC-tagged zymosan targets were engulfed significantly faster and in higher amounts compared to untreated controls **(A, C, E)**. mCRP added in the same concentration showed no significant effects on the phagocytosis in the assessed subtypes **(B, D, F)**. All results are presented as means and S.E.M. and P values were calculated with ANOVA and *Tukey post hoc* test, n = 6, ^#^not significant, * and ** P < 0.05 and < 0.01.

Phagocytosis of Zymosan was evaluated as another established target in phagocytosis assays. pCRP enhanced the phagocytosis especially for late time points in all three leukocyte subtypes (**Figures 5A, C, E**, * *P* < 0.05, ** *P* < 0.01 and # *P* > 0.05). However, mCRP did not influence the phagocytosis of Zymosan whatsoever (**Figures 5B, D, F**).

### The Complement System Is Essential for Phagocytosis in All Leukocyte Subsets

**Figure 6:**
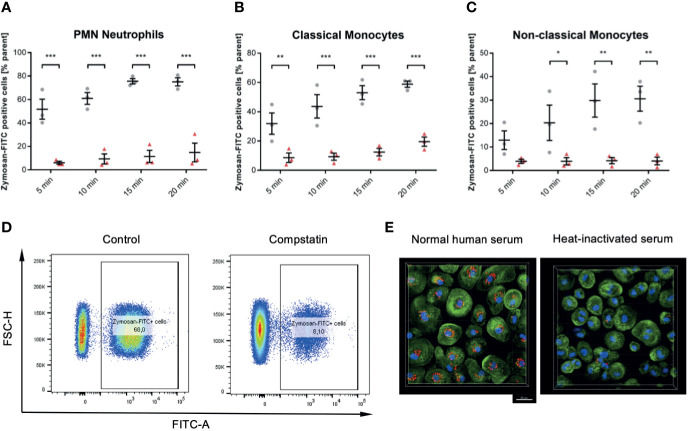
Inhibition of the C3 convertase by Compstatin reduces phagocytosis of zymosan dramatically in whole blood leukocytes. Whole blood treated with 50 µM Compstatin 15 min before the exposure to the phagocytosis targets presented significantly reduced phagocytosis in all assessed subtypes. Compstatin-treated neutrophils showed massively reduced phagocytotic activity at any given time point (**A**, *** P < 0.001). In classical monocytes **(B)**, the difference to an untreated control for Zymosan-positive cells was the lowest at 5 min time-point (** P < 0.01), with only minor change over time in the Compstatin-treated specimen, resulting in an increase of difference (*** P < 0.001 for 10, 15, and 20 min). Non-classical monocytes **(C)** showed a similar tendency with the 5 min time-point not significant (P > 0.05), but the following time-points with increasing significant differences to the untreated control (* P < 0.05 for 10 min and ** P < 0.01 for 15 and 20 min). The results are presented as means and S.E.M. and P values were calculated with ANOVA and *Tukey post hoc* test, n = 3, *, ** and *** P < 0.05, < 0.01 and < 0.001, respectively. **(D)** shows representative results from phagocytosis by PMNs at the 15 min time-point **(E)**. The overall substantial role in the phagocytosis of zymosan-particle by monocytes was verified by confocal fluorescence microscopy in normal human serum and heat-inactivated serum, respectively. After 45 min of phagocytosis, cells without functioning complement showed significantly less engulfed zymosan (red). Scale bar indicates 20 µm.

Compstatin inhibits the C3 convertase and therefore inhibits the activation of the classical complement pathway at the most upstream point within the complement system. In whole blood samples treated with 50 µM Compstatin phagocytosis of Zymosan was reduced significantly in all three assessed leukocyte subsets (*P* < 0.05 *, < 0.01 **, < 0.001 ***, < 0.0001 ****), with exception for non-classical monocytes at the 5 min time-point (**Figures 6A–C**). The phagocytotic active cells were clearly distinguishable when treated with Compstatin as demonstrated representatively by the final gate of neutrophils at 15 min in the used flow cytometry gating strategy (**Figure 6D**). This substantial role of complement in phagocytosis was verified by confocal fluorescence microscopy of zymosan-particles (red) in normal human serum and heat-inactivated serum, respectively (**Figure 6E**).

### Neutrophil Phagocytosis Efficacy Depends More on Receptor C5aR1 Compared to Monocyte Subsets

**Figure 7:**
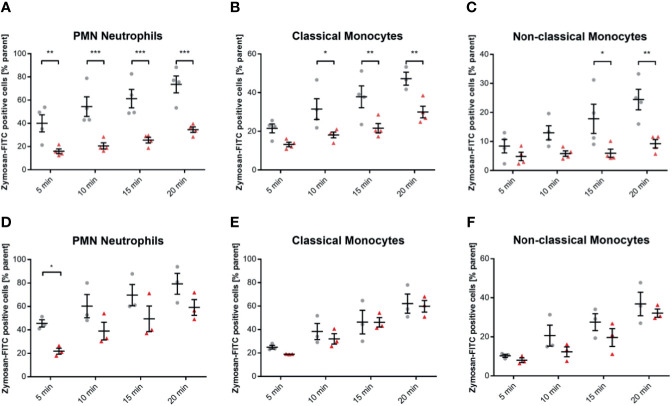
Inhibition of C5aR1 impedes phagocytosis of zymosan dramatically in whole blood leukocytes, while OmCI does not. Blood was treated with 10 µg/mL C5aR1 antagonist PMX53 **(A–C)** and C5 inhibitor OmCI **(D–F)**, respectively. 15 min before the exposure to the phagocytosis targets, the relevant inhibitor was added, and phagocytosis was assessed subset-specifically. PMX53-treated neutrophils **(A)**, classicals **(B)**, and non-classicals **(C)** showed reduced phagocytotic activity. However, neutrophils were affected by C5aR1 antagonism by PMX53 in particular (*P* < 0.05 *, *P* < 0.01 **, < 0.001 ***, **(A)**. PMX53 reduced phagocytosis significantly in both monocyte subsets with exception for early time points **(B, C)**. However, we did not find the C5 inhibitor OmCI to reduce the phagocytotic activity in any subtype and time point significantly except for the 5 min time-point in neutrophils **(D–F)**.

Human blood samples treated with 10 µg/mL of the specific C5a receptor 1 antagonist PMX53 showed reduced phagocytotic activity against Zymosan targets by neutrophils in particular (*P* < 0.01 **, < 0.001 ***, **Figure 7A**). However, PMX53 reduced phagocytosis significantly in both monocyte subsets with exception for early time points (**Figures 7B, C**). However, we did not find the C5 inhibitor OmCI to reduce the phagocytotic activity in any subtype and time point significantly except for the 5 min time-point in neutrophils (**Figures 7D–F**).

### Confocal Fluorescence Microscopy Confirms pCRP-Opsonization of *S. pneumoniae* and Complement Activation as Two Crucial Parts of Phagocytosis

**Figure 8:**
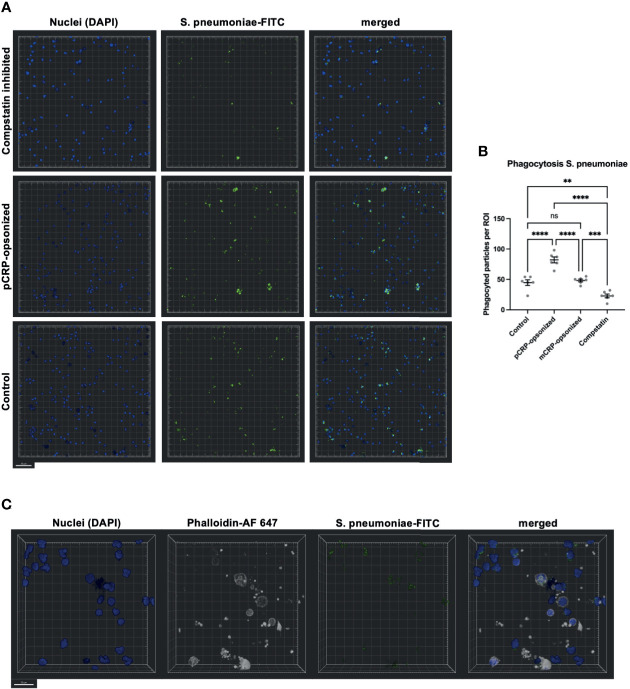
Phagocytosis in whole blood is impeded by inhibition of complement and enhanced by pCRP-opsonization as measured by confocal fluorescence microscopy. Blood was treated as described above. 15 min before the exposure to the phagocytosis targets, the relevant inhibitor was added, and phagocytosis was assessed at 20x magnification. Shown are representative results for pCRP-opsonized S. pneumoniae, Compstatin inhibited cells, and the untreated control group, respectively. Compstatin-treated phagocytic leukocytes (DAPI, blue) showed significantly reduced activity in the phagocytosis of S. pneumoniae-FITC targets (green). When pCRP was added to the targets first, the number of phagocytized particles was significant higher. Scale bar indicates 50 µm **(A)**. However, mCRP added to the targets showed no difference to the unstimulated control. Results are presented as means and S.E.M. and P values were calculated with ANOVA and *Tukey post hoc* test, n = 6, ns, not significant, ** P < 0.01, *** and **** < 0.001, and < 0.0001 **(B)**. Only FITC-positive targets (green) in direct proximity to the nucleus (blue) and surrounded by F-actin (white) were assumed to be phagocyted particles. Scale bar indicates 20 µm **(C)**.

Blood was treated as described above and phagocytosis exemplarily assessed for S. pneumoniae by confocal fluorescence microscopy (**Figure 8**). Again, Compstatin reduced the phagocytosis activity significantly (** P < 0.01 *vs*. Control). pCRP-opsonized particles were phagocytized in higher numbers, while mCRP treatment of the particles prior to exposure to whole blood showed no significant differences compared to an untreated control (**** P < 0.0001 and ns P > 0.05, **Figure 8B**). S. pneumoniae-FITC targets were counted as phagocytized when in direct proximity to the nucleus and surrounded by f-actin (**Figure 8C**).

## Conclusions

In this study we present novel findings regarding the proinflammatory effects of CRP in innate immunity host defense mechanisms. We found CRP to have a major role in scavenging common pathogens and further consider CRP a rewarding target in anti-inflammatory therapy. The following findings support this: (1) human whole blood samples assessed in flow cytometry are a convenient option for the quantitative detection of innate host defense mechanisms as described by our methods, (2) CRP enhances ROS generation in neutrophils and both classical and non-classical monocytes in a conformation-specific manner. mCRP, but not pCRP induces respiratory bursts in all three subsets, (3) ROS generation in classical monocytes and neutrophils relies on the activation of the complement system, but not on C5aR1 receptor activation. The appropriate stimulation of phagocytic leukocytes causes the assembly of the nicotinamide adenine dinucleotide phosphate oxidase complex (NADPH oxidase), which subsequently produces the cytotoxic agent superoxide from oxygen, a process referred to as the respiratory burst (15–18). During sepsis an excess of ROS production by neutrophils and monocytes exacerbates host tissue injury and causes endothelial dysfunction leading to an immunopathological response. The evaluation of the respiratory burst is based on dihydroethidium (DHE). DHE is a fluorogenic dye for detecting superoxide, which oxidizes DHE, a non-fluorescent compound, to form 2-hydroxyethidium (2-OH-E+). 2-OH-E+ itself is then detectable at 590-620 nm by fluorimetry after excitation with 500-530 nm. We tested the established complement system inhibitors Compstatin, PMX53, and OmCI to validate the role of complement in these host defense mechanisms. The importance of a functioning NADPH oxidase complex is clinically apparent in patients suffering from chronic granulomatous disease, an immunodeficiency due to non-functioning NADPH oxidase. Patients exhibit increased susceptibility to invading pathogens (19). We hereby describe a novel and convenient method for the quantitative detection of basic innate host defense mechanisms in small human whole blood samples.

CRP represents a key factor in amplifying the response to tissue damage: Acting as a main initiator of the classical complement pathway, CRP aggravates the inflammatory response mainly in a complement-dependent manner. Complement C5a fragments constitute a cleavage product on the final common pathway of this basic defense mechanism. *Via* complement C5a receptor, this anaphylatoxin exerts a strong inflammatory effect on granulocytes and monocytes (5). Our group has recently shown that pentameric CRP (pCRP) localized to disturbed membranes in injured tissue and undergoes conformational changes leading to complement activation *via* newly expressed binding sides for C1q (6). In polytraumatized patients neutrophils can generate a vast amount of ROS and form an acidic microenvironment dependent on complement activation and C5a (20).

CRP was found to facilitate the phagocytosis of S. pneumoniae and Zymosan, but not E. coli. Most interesting, the serotype 27 of S. pneumoniae expresses phosphocholine (PCh) as a part of its capsule (21, 22). The capsular PCh makes serotype 27 most accessible to the opsonization by CRP and thus, this serotype of S. pneumoniae is regarded as nonpathogenic (23, 24). In contrast, E. coli recognition and immune responses are based mostly on toll-like receptors and rapid opsonization by complement C1q, mannose-binding lectin, C4b, C3b/iC3b, and immunoglobulins (25, 26). CRP plays a minor role in the opsonization of the gram-negative bacterium E. coli (27). However, flow cytometry based evaluation of phagocytosis is prone to overestimation of the phagocytosis activity due to missing differentiation of bound and phagocytized particles. Confocal fluorescence microscopy was used to partially compensate these shortcomings. We found the same tendencies in the evaluation of phagocytosis in both assays.

Circulating platelets play a major role in host defense (28). As described before (15), disturbed and activated surfaces of thrombocytes serve as a bio-activating source of the conformational change in CRP and neo-epitope expression for C1q binding (6). The pro-inflammatory potential of pCRP binding to activated membranes and its consecutive generation of pCRP*/mCRP has been proven before in both *in vitro* and *in vivo (* 6, 7, 18).

## Data Availability Statement

The original contributions presented in the study are included in the article/**Supplementary Material**. Further inquiries can be directed to the corresponding author.

## Ethics Statement

This study was carried out in accordance with the recommendations of the animal ethic committee of the University of Freiburg Medical Center, Germany. The patients/participants provided their written informed consent to participate in this study.

## Author Contributions

JZ drafted the script and concept and designed and planned all experiments. BB and MF contributed the main part of acquiring the experimental data. JK and DB revised the manuscript. EK and MH-L helped to interpret the complement-specific data. KP and DB served as important advisors and critically revised the manuscript. SE is the supervisor of the described work and the principal investigator. MH-L and SE contributed equally to this work. All authors contributed to the article and approved the submitted version.

## Funding

This work was supported by personal grants to SE from the German Research Foundation (DFG) EI 866/1-1, EI 866/1-2, EI 866/5-1, and EI 866/10-1. SE is a Heisenberg Professor of the DFG (EI 866/4-1 and EI 866/9-1). The confocal microscope "Zeiss LSM 980" used in this study was funded by the DFG (INST 39/1137-1FUGG).

## Conflict of Interest

The authors declare that the research was conducted in the absence of any commercial or financial relationships that could be construed as a potential conflict of interest.

## Publisher’s Note

All claims expressed in this article are solely those of the authors and do not necessarily represent those of their affiliated organizations, or those of the publisher, the editors and the reviewers. Any product that may be evaluated in this article, or claim that may be made by its manufacturer, is not guaranteed or endorsed by the publisher.

## Supplementary Material

Supplementary Figure 1 Gating strategy for ROS in human whole blood samples. Flow cytometry-based investigation of ROS generation in neutrophils and both primary subtypes of monocytes. Cell suspensions were first assessed by FSC-A and SSC-A distribution. A threshold was set to 35,000 to reduce noise by cell debride and platelets. Neutrophils (PMN, top row) were gated forward and plotted FSC-area against FSC-height to exclude doublets. Single cells were further assessed for CD16 expression (PE-Cy7 fluorescence) and CD16 high positive cells were assumed to be neutrophils. Emission of 2-hydroxyethidium (2-OH-E+) was finally acquired in this group and regarded as a surrogate for ROS generation. For classical (middle row) and non-classical monocytes (bottom row), doublets were excluded as described above and then assessed for CD16 and CD14 expression. Classical monocytes (CD14++, CD16-) were gated forward and analyzed for HLA-DR expression. The HLA-DR dim and positive population was assumed to be classical monocytes and ROS generation was assessed as described above. Non-classical monocytes (CD14+CD16++) were gated forward and analyzed for HLA-DR expression. The HLA-DR positive population was assumed to be classical monocytes and ROS generation was assessed as described above.

Supplementary Figure 2 ROS detection in leukocytes as measured by DHE in whole blood samples. Human whole blood samples were incubated with reagents as described above. Shown is the ROS generation in three leukocyte subsets as analyzed after RBC for DHE fluorescence as a surrogate. Cell suspensions were assessed by FSC-A and SSC-A distribution and a threshold was set to 35,000 to exclude debris. Lymphocytes were gated forward, and doublets were excluded. CD14 and HLA-DR negative single cells were assumed to be lymphocytes and were assessed for DHE fluorescence **(A)**. Lymphocytes were set as a negative control for ROS production. Neutrophils and classical monocytes show a shift in the DHE specific channel (ex 488 nm, em 575/26 nm) when stimulated with LPS and mCRP (blue histograms) when compared to an unstimulated control (red histogram). However, both LPS and mCRP failed to have an effect on DHE fluorescence in lymphocytes **(B)**.

Supplementary Figure 3 ROS detection with CellROX Deep Red in whole blood samples. Human whole blood samples were treated as described above. Shown is the ROS generation in three relevant leukocyte subsets. CellROX Deep Red was adapted for the detection of ROS as described by the producer. Cell suspensions were assessed by FSC-A and SSC-A distribution and a threshold was set to 35,000 to exclude debris. Neutrophils were gated as described above. Classicals and non-classicals were gated as described above and additionally, PE-positive cells were excluded from analysis **(A)**. CellROX Deep Red specific fluorescence was measured in neutrophils and both monocyte subtypes. Neutrophils and both classicals and non-classicals showed a significant shift when stimulated with LPS and mCRP (blue histograms) compared to an unstimulated control (red histogram). Representative results are shown. **(C)** All results are presented as means and S.E.M. and P values were calculated with ANOVA and *Tukey post hoc* test, n=3, ns not significant, ** P < 0.01, *** and **** < 0.001, and < 0.0001.

Supplementary Figure 4 The described gating strategy allows the precise assessment of leukocyte subtype-specific phagocytosis of common targets in whole blood samples. Human whole blood samples were incubated with the three common phagocytosis targets Zymosan, S. pneumoniae and E. coli. Shown is the phagocytosis of heat-killed S. pneumoniae targets as analyzed after RBC for FITC-tagged phagocytosis targets at the time-points described (representative image for time-point 15 min shown). The target cells tested are three typical pathogens: two bacterial (S. pneumoniae and E. coli) and one fungal origin (Zymosan). Cell suspensions were assessed by FSC-A and SSC-A distribution and a threshold was set to 50,000. PMNs were gated forward, and doublets were excluded. CD16 high positive single cells were assumed to be neutrophils and were assessed for FITC fluorescence. Phagocytosis positive gate was adjusted on a target-free control (not shown). Classicals (middle row) and non-classicals (bottom row) were first reduced to singlets as described above and then assessed for CD16 and CD14 expression. CD14++CD16- and CD14+CD16++, respectively, were gated forward and analyzed for FITC positive cells as described above **(A)**. By this method phagocytosis was distinguishable both at different time points and subset- and target-specific **(B, C)**.
