# Supplementary figures and images for: CRP Enhances the Innate Killing Mechanisms Phagocytosis and ROS Formation in a Conformation and Complement-Dependent Manner
Source: Front Immunol. 2021 Aug 10;12:721887. doi: 10.3389/fimmu.2021.721887 (PMC8383111; doi:10.3389/fimmu.2021.721887)

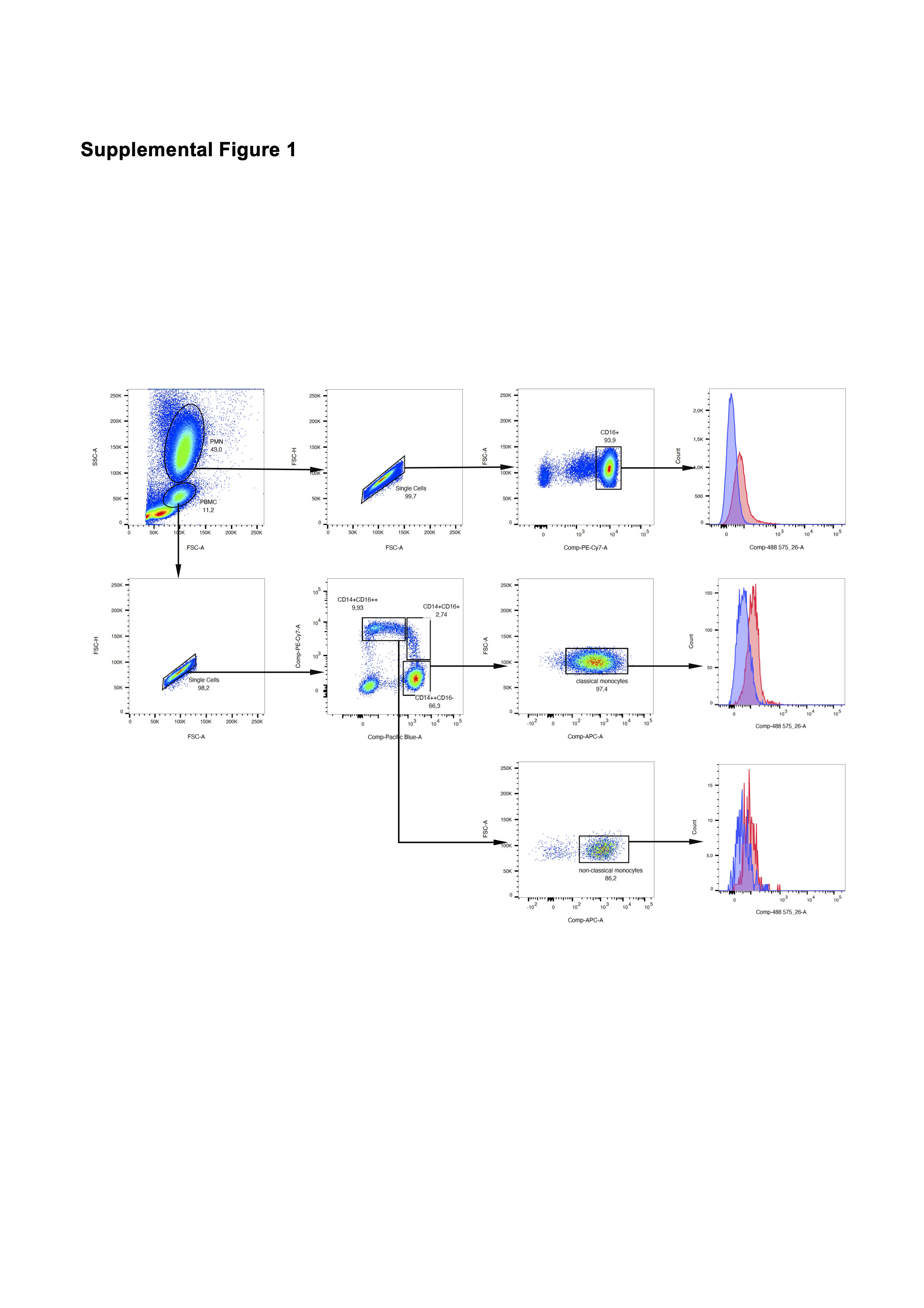

Supplement: Supplementary Figure 1 — Gating strategy for ROS in human whole blood samples. Flow cytometry-based investigation of ROS generation in neutrophils and both primary subtypes of monocytes. Cell suspensions were first assessed by FSC-A and SSC-A distribution. A threshold was set to 35,000 to reduce noise by cell debride and platelets. Neutrophils (PMN, top row) were gated forward and plotted FSC-area against FSC-height to exclude doublets. Single cells were further assessed for CD16 expression (PE-Cy7 fluorescence) and CD16 high positive cells were assumed to be neutrophils. Emission of 2-hydroxyethidium (2-OH-E+) was finally acquired in this group and regarded as a surrogate for ROS generation. For classical (middle row) and non-classical monocytes (bottom row), doublets were excluded as described above and then assessed for CD16 and CD14 expression. Classical monocytes (CD14++, CD16-) were gated forward and analyzed for HLA-DR expression. The HLA-DR dim and positive population was assumed to be classical monocytes and ROS generation was assessed as described above. Non-classical monocytes (CD14+CD16++) were gated forward and analyzed for HLA-DR expression. The HLA-DR positive population was assumed to be classical monocytes and ROS generation was assessed as described above. [file Image_1.tif]

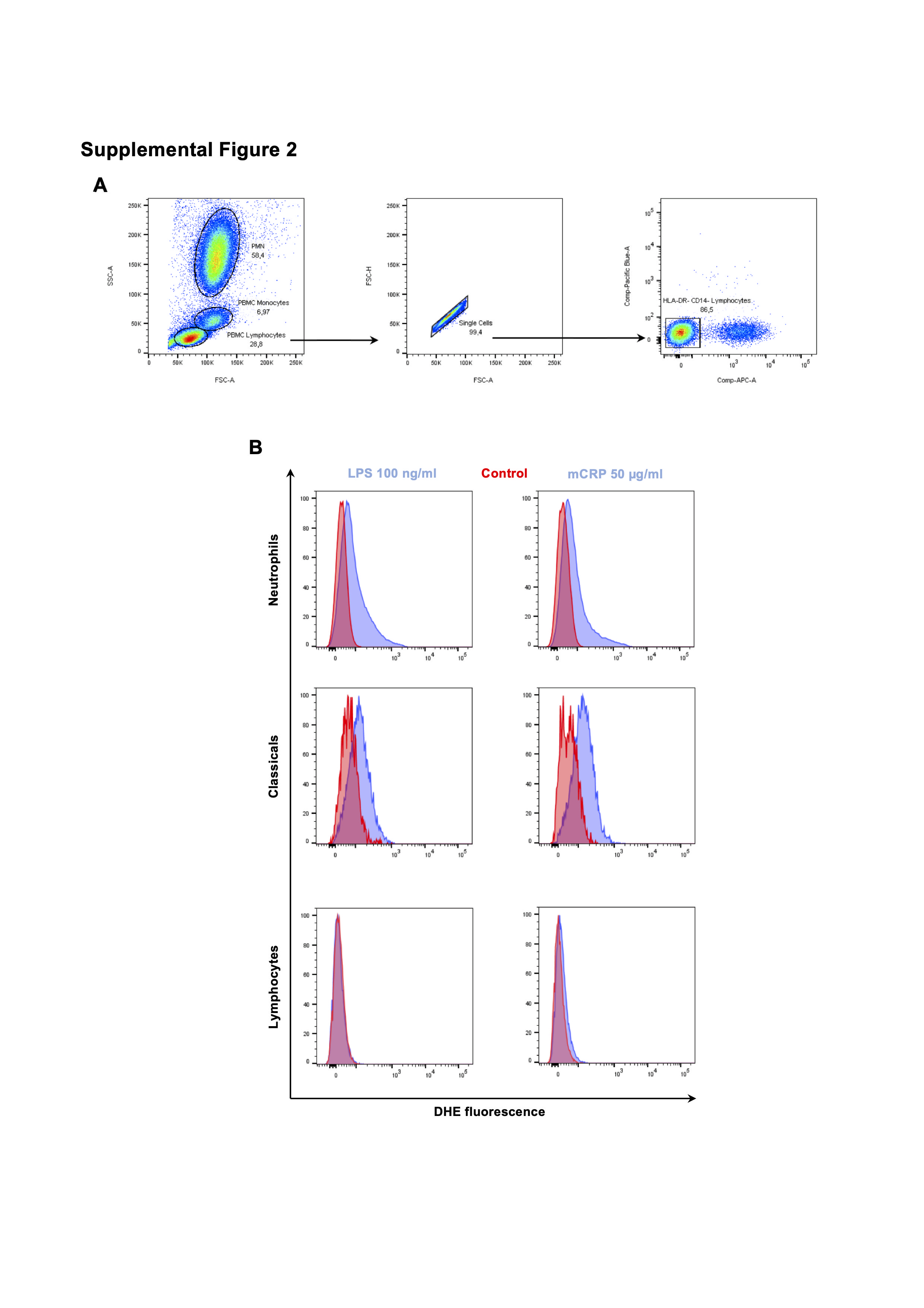

Supplement: Supplementary Figure 2 — ROS detection in leukocytes as measured by DHE in whole blood samples. Human whole blood samples were incubated with reagents as described above. Shown is the ROS generation in three leukocyte subsets as analyzed after RBC for DHE fluorescence as a surrogate. Cell suspensions were assessed by FSC-A and SSC-A distribution and a threshold was set to 35,000 to exclude debris. Lymphocytes were gated forward, and doublets were excluded. CD14 and HLA-DR negative single cells were assumed to be lymphocytes and were assessed for DHE fluorescence (A). Lymphocytes were set as a negative control for ROS production. Neutrophils and classical monocytes show a shift in the DHE specific channel (ex 488 nm, em 575/26 nm) when stimulated with LPS and mCRP (blue histograms) when compared to an unstimulated control (red histogram). However, both LPS and mCRP failed to have an effect on DHE fluorescence in lymphocytes (B). [file Image_2.tif]

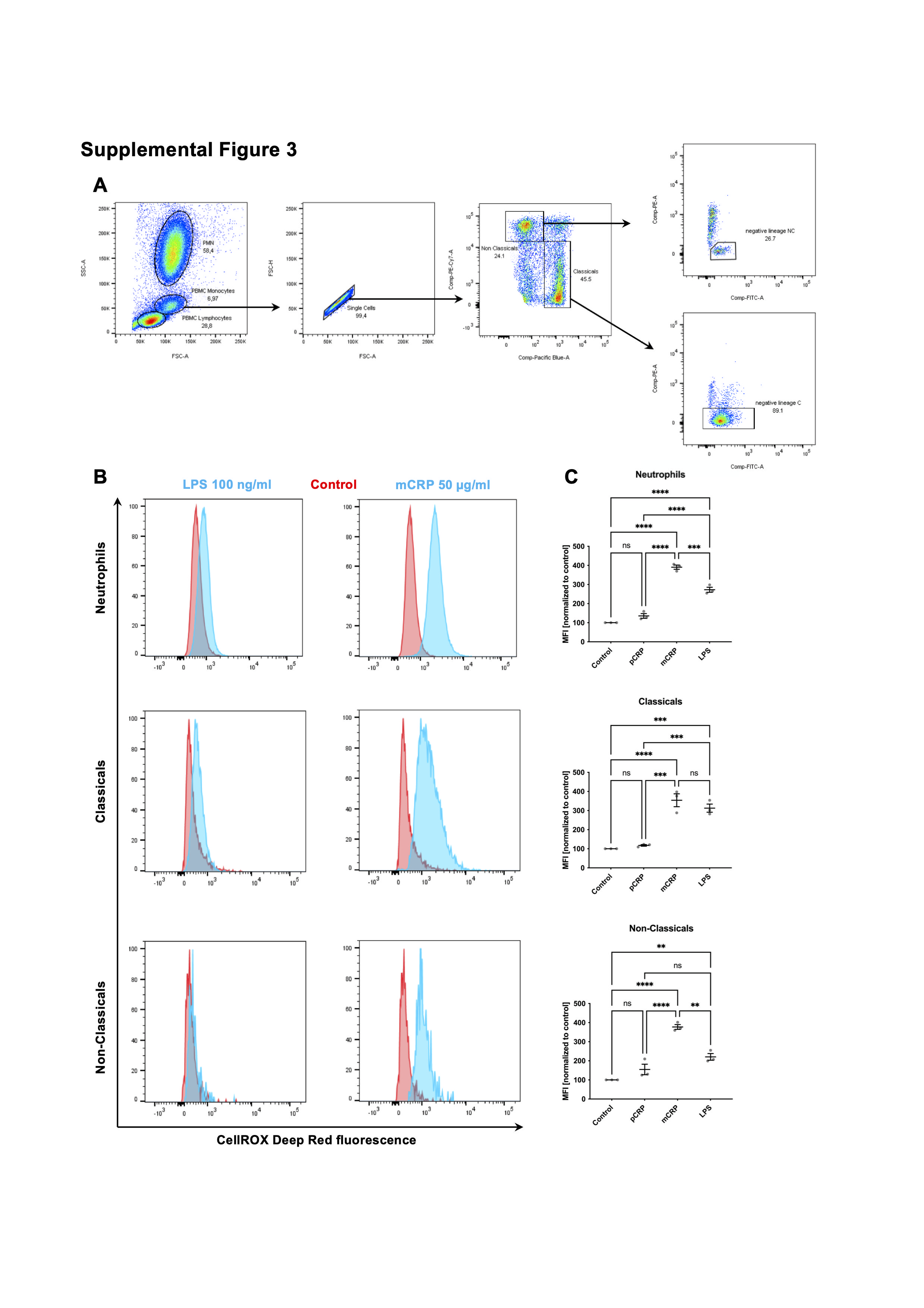

Supplement: Supplementary Figure 3 — ROS detection with CellROX Deep Red in whole blood samples. Human whole blood samples were treated as described above. Shown is the ROS generation in three relevant leukocyte subsets. CellROX Deep Red was adapted for the detection of ROS as described by the producer. Cell suspensions were assessed by FSC-A and SSC-A distribution and a threshold was set to 35,000 to exclude debris. Neutrophils were gated as described above. Classicals and non-classicals were gated as described above and additionally, PE-positive cells were excluded from analysis (A). CellROX Deep Red specific fluorescence was measured in neutrophils and both monocyte subtypes. Neutrophils and both classicals and non-classicals showed a significant shift when stimulated with LPS and mCRP (blue histograms) compared to an unstimulated control (red histogram). Representative results are shown. (C) All results are presented as means and S.E.M. and P values were calculated with ANOVA and Tukey post hoc test, n=3, ns not significant, ** P < 0.01, *** and **** < 0.001, and < 0.0001. [file Image_3.tif]

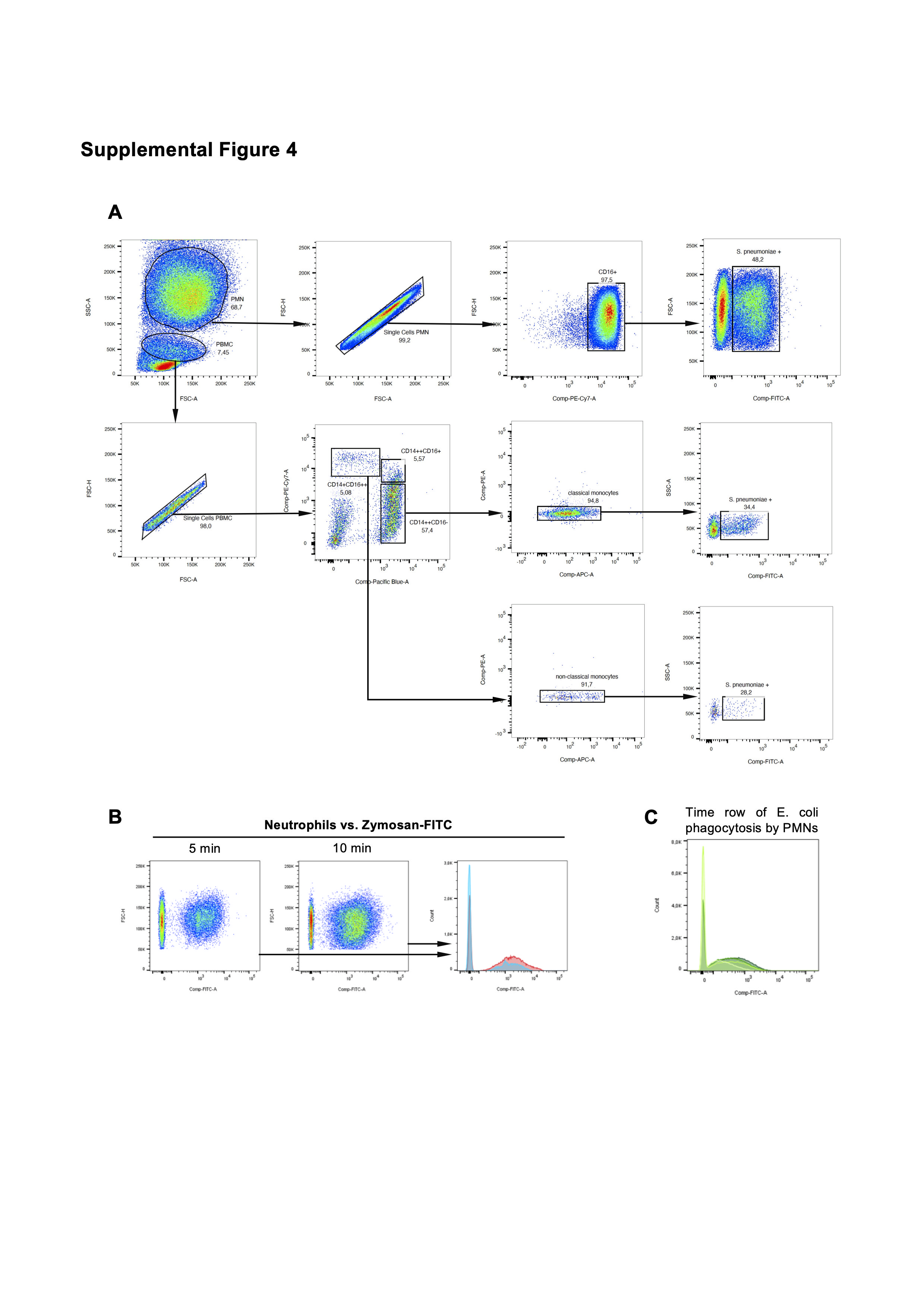

Supplement: Supplementary Figure 4 — The described gating strategy allows the precise assessment of leukocyte subtype-specific phagocytosis of common targets in whole blood samples. Human whole blood samples were incubated with the three common phagocytosis targets Zymosan, S. pneumoniae and E. coli. Shown is the phagocytosis of heat-killed S. pneumoniae targets as analyzed after RBC for FITC-tagged phagocytosis targets at the time-points described (representative image for time-point 15 min shown). The target cells tested are three typical pathogens: two bacterial (S. pneumoniae and E. coli) and one fungal origin (Zymosan). Cell suspensions were assessed by FSC-A and SSC-A distribution and a threshold was set to 50,000. PMNs were gated forward, and doublets were excluded. CD16 high positive single cells were assumed to be neutrophils and were assessed for FITC fluorescence. Phagocytosis positive gate was adjusted on a target-free control (not shown). Classicals (middle row) and non-classicals (bottom row) were first reduced to singlets as described above and then assessed for CD16 and CD14 expression. CD14++CD16- and CD14+CD16++, respectively, were gated forward and analyzed for FITC positive cells as described above (A). By this method phagocytosis was distinguishable both at different time points and subset- and target-specific (B, C). [file Image_4.tif]
